# Body Mass Index Trajectories and Healthcare Utilization in Young and Middle-aged Adults

**DOI:** 10.1097/MD.0000000000002467

**Published:** 2016-01-15

**Authors:** Muhamad Y. Elrashidi, Debra J. Jacobson, Jennifer St. Sauver, Chun Fan, Brian A. Lynch, Lila J. Finney Rutten, Jon O. Ebbert

**Affiliations:** From the Department of Medicine (MYE, JOE); Department of Health Sciences Research (DJJ, JSS, LJFR, CF); Department of Community Pediatric and Adolescent Medicine (BAL); and Robert D. and Patricia E. Kern Center for the Science of Health Care Delivery, Mayo Clinic, Rochester, MN (LJFR, JOE).

## Abstract

The obesity epidemic is a significant public health issue with adverse impact on health and costs. Applying a life-course perspective to obesity may advance our understanding of the influence of obesity over time on patterns of healthcare utilization in young and middle-aged United States (US) adults.

We identified baseline body mass index (BMI) and BMI trajectories, and assessed their association with outpatient visits, emergency department (ED) visits, and hospitalizations in a well-defined population of young and middle-aged US adults.

Using the Rochester Epidemiology Project resources, we conducted a retrospective cohort study of adults (N = 23,254) aged 18 to 44 years, with at least 3 BMI measurements, residing in Olmsted County, MN from January 1, 2005 through December 31, 2012.

We observed that 27.5% of the population was obese. Four BMI trajectories were identified. Compared to under/normal weight, obese class III adults had higher risk of outpatient visits (adjusted rate ratio [RR], 1.86; 95% confidence intervals [CIs], 1.67–2,08), ED visits (adjusted RR, 3.02; 95% CI, 2.74–3.34), and hospitalizations (adjusted RR, 1.67; 95% CI, 1.59–1.75). BMI trajectory was positively associated with ED visits after adjustment for age, sex, race, and Charlson Comorbidity Index (*P* < 0.001 for trend).

Among young and middle-aged US adults, baseline BMI is positively associated with outpatient visits, ED visits, and hospitalizations, while BMI trajectory is positively associated with ED visits. These findings extend our understanding of the longitudinal influence of obesity on healthcare utilization in early to mid-adulthood.

## INTRODUCTION

The obesity epidemic remains an urgent public health crisis with significant impact on morbidity, mortality, and healthcare costs.^1^ More than one-third of all Americans are obese (body mass index [BMI] ≥30 kg/m^2^) and the prevalence of obesity in the United States (US) is on track to rise to 50% by 2030.^2,3^ The adverse health consequences of obesity include cardiovascular disease, hypertension, dyslipidemia, diabetes mellitus, sleep apnea, and psychiatric comorbidities including depression.^4^ Obesity contributes to at least 162,000 deaths each year across all ages and can shorten life expectancy by nearly a decade in young and middle-aged adults.^4–6^ In addition, medical costs in the US related to obesity are $210 billion per year^7^ and projected to increase by an additional $48 to $66 billion by 2030.^3^

Underlying the disparities in medical costs between obese and nonobese individuals are different patterns of healthcare utilization. Focus on healthcare utilization and costs in the elderly is expected given that younger adults tend to use health services comparatively less than older adults.^8^ However, similar to the concentrated healthcare costs and utilization observed among older adults, 5% of 18- to 44-year olds account for more than 50% of all healthcare expenditures in their age group.^9^ In addition, adults typically gain most of their weight in their early decades.^10^ Adults experiencing higher weight gain in young adulthood are likely to have sustained, higher weight later in life.^11^ Obesity and obesity-related conditions are likely contributors to increased healthcare utilization as cross-sectional studies in the US have demonstrated that higher BMI is associated with higher rates of outpatient clinic visits, emergency department (ED) visits, and hospitalizations.^12–14^ However, analyses based on cross-sectional measures of BMI fail to capture the potential influence of weight change over time.^15^ BMI increase over the life course, particularly in early to middle adulthood, may uniquely contribute to increased healthcare utilization.

A longitudinal perspective allows for a better understanding of the influence of obesity over the life course and provides a means to identify differential health outcomes and healthcare experiences in population subgroups.^16–20^ In recent years, researchers applying this approach have characterized the underlying heterogeneity of BMI trajectories within several age-defined cohorts.^11,16–19,21^ BMI trajectories have been shown to be associated with risk for diabetes, hypertension, and mortality.^20,22,23^ Although a few studies have recently begun to examine the life-course impact of obesity on healthcare utilization, these investigations were conducted outside the US and/or focused on childhood cohorts.^24–26^ In addition, none of these studies assessed the underlying heterogeneity of BMI trajectories in order to evaluate if BMI trajectory influences healthcare utilization. As a result, the relationship between longitudinal changes in BMI and healthcare utilization, particularly among US adults who bear a considerable obesity-related burden, remains unclear. To address this gap, we examined the associations of baseline BMI and BMI trajectories with outpatient clinic visit days, ED visits, and hospitalizations among young and middle-aged adults.

## METHODS

### Study Population

We identified all residents of Olmsted County, Minnesota (MN), aged 18 to 44 years on January 1, 2005 (N = 54,962) excluding those who had not given permission to use their medical research, through the Rochester Epidemiology Project (REP).^27,28^ Most medical care in Olmsted County, MN is provided by 3 healthcare institutions whose electronic health records (EHRs) are linked together through the REP medical records linkage system to enable research.^27,28^ The description of this medical records linkage system, which captures virtually all residents of Olmsted County, and its capacity for providing an effective sampling framework for similar studies has been previously described.^29^ The REP population estimates reflect the 2005 US census estimates with a slight over count of individuals 20 to 29 years of age.^30^

At least 1 BMI measurement was available for 32,075 individuals in the targeted age range, representing 58% of total Olmsted County 18- to 44-year-old adults. At least 3 BMI measurements per person are recommended for trajectory analyses and therefore, analyses were completed only on those with at least 3 BMI measurements (N = 23,254). The characteristics of those with at least 3 BMI measurements were similar to the total population of Olmsted County, MN in 2005, with slightly more females and whites (sample population: 36.9% male, 87.3% white, median age 33.2; total population: 46.5% male, 79.6% white, median age 30.8). The Mayo Clinic and Olmsted Medical Center Institutional Review Boards approved this study.

### Exposure Variables

We obtained electronic height and weight data measured between January 1, 2005 and December 31, 2012 for our study population. Height and weight measurements were available electronically from a single, large, multisite healthcare provider. Weights of pregnant women were excluded for the last 6 months of pregnancy and the first 3 months of postpartum. EHRs were reviewed for potential outliers to exclude obvious data errors. A BMI was calculated for each weight measurement (kg/m^2^) using the most recently measured height. BMI was categorized into 5 groups based on the classification criteria of the World Health Organization.^31^ We categorized underweight as BMI < 18.5 kg/m^2^, normal weight as BMI (18.5–25.5) kg/m^2^ and the remaining groups as follows: overweight (25–29.9 kg/m^2^), obese class I (30–34.9 kg/m^2^), obese class II (35–39.9 kg/m^2^), and obese class III (≥40 kg/m^2^).^31^ Age, sex, and race were considered as potential confounding variables. We calculated a Charlson Comorbidity Index (CCI) based on ICD-9 codes for each patient to use as a surrogate measure of patient medical complexity.^32^

### Outcome Variables

We identified the dates of all outpatient visits, ED visits, hospitalizations, and dates of death from January 1, 2005 through December 31, 2012 for our study population. The distributions of the visits were assessed and ED visits were categorized as 0, 1 to 2, and 3 or more visits. Hospitalizations were categorized as 0, 1, and 2 or more hospitalizations. The number of outpatient visit days was considered as a continuous variable.

### Statistical Analysis

Baseline characteristics of the population by initial BMI categories were compared across BMI groups using a χ^2^ test for categorical variables and Kruskal–Wallis test for continuous variables. The rates per person year of outpatient visit days, ED visits, and hospitalizations during follow-up were determined. Associations between baseline BMI group and rate of outpatient visits, ED visits, and hospitalizations were assessed using negative binomial regression and are presented as rate ratios (RRs) with 95% confidence intervals (CIs). Those in the normal weight category served as the referent group. Multivariable models were used to adjust for the effect of sex, race, and age (continuous). Additional models were used to further adjust for the CCI. Homogeneous clusters of BMI trajectories were identified for those with 3 or more BMI measurements (N = 23,261) using the Kml R package which uses K-means, a nonparametric hill-climbing algorithm, to identify distinct clusters and assign individuals to unique clusters.^33^ The Calinski and Harabatz criterion was used to determine the optimum number of clusters.^33^ The mean of all individual trajectories in a cluster was plotted to summarize the overall change over time for each group. Trajectories were assessed for males and females separately, but since trajectories were similar, only the combined results are presented. Similar trajectories were found using *proc traj* in SAS version 9.3 (SAS Institute, Cary, NC). Negative binomial regression models were used to assess the association between BMI trajectory and rate of outpatient visit days, ED visits, and hospitalizations over the same time period and are presented as adjusted RR with 95% CIs. Multivariable models were used to adjust for the effect of sex, race, age (continuous), and baseline BMI class. There were few numbers of deaths in this age group to allow for an assessment of the association between BMI groups and mortality. Analyses were performed using SAS version 9.3 (SAS Institute) and R version 2.14.0 (The R Foundation for Statistical Computing).

## RESULTS

Characteristics of the population by baseline BMI level are shown in Table 1. Overall, 27.5% (N = 6396) of the population was obese. At baseline, 1.7% were underweight, 39.9% were normal weight, 30.8% were overweight, 16.0% were obese class I, 6.9% were obese class II, and 4.6% were obese class III. Individuals in obese class III were slightly older and had more comorbidity compared to those who were under/normal weight. Whites, blacks, and females were more frequently obese. In addition, increasing frequency of outpatient visit days, ED visits, and hospitalizations were observed with each BMI class among those who were obese (Table 1).

**TABLE 1:**
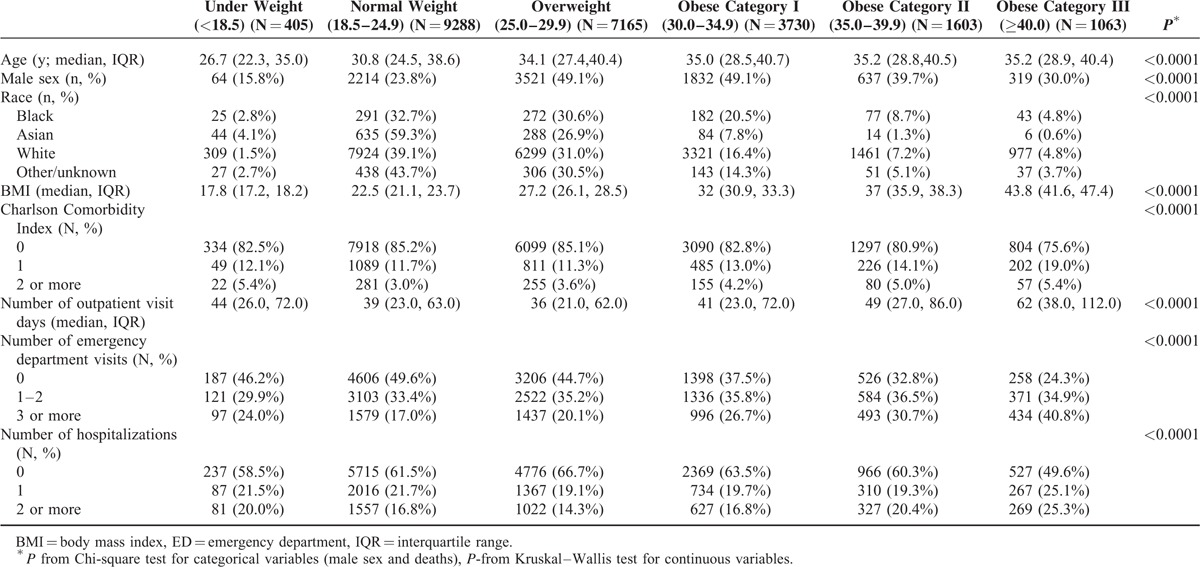
Characteristics of the 2005 Olmsted County, Minnesota Population (18–44 Years) With At Least Three Body Mass Index Measure (N = 23,254)

The rate of outpatient visit days, ED visits, and hospitalizations increased with BMI category over a median follow-up of 7.2 years (Table 2). The mean rate of outpatient visit days increased from 8.2 in those with normal weight to 13.6 in those in obese class III (adjusted RR, 1.86; 95% CI, 1.67–2.08; Table 3). Similarly, the mean rate of ED visits increased from 0.3 per person year for those with normal weight to 0.7 per person year for obese class III adults (adjusted RR, 3.02; 95% CI, 2.74–3.34; Table 3). The mean rate of hospitalizations increased from 0.1 per person year for those with normal weight to 0.2 per person year for those in obese class III (adjusted RR, 1.67; 95% CI, 1.59, 1.75; Table 3). Additionally, there was an increased rate of outpatient visit days (RR, 1.26; 95% CI, 1.20, 1.32), ED visits (RR, 1.58; 95% CI, 1.51, 1.65), and hospitalizations (RR, 1.20; 95% CI, 1.18, 1.23), for those who were underweight compared to those who were normal weight (Table 3). Results were similar with further adjustment for comorbidity using the CCI (data not shown). Similar results were also seen when a 2-part model was applied to ED visits and hospitalizations. There was an increased association of any utilization with increasing BMI category, compared to no utilization and a significant, but attenuated rate of increased utilization with increasing BMI level for those with utilization (data not shown).

**TABLE 2:**
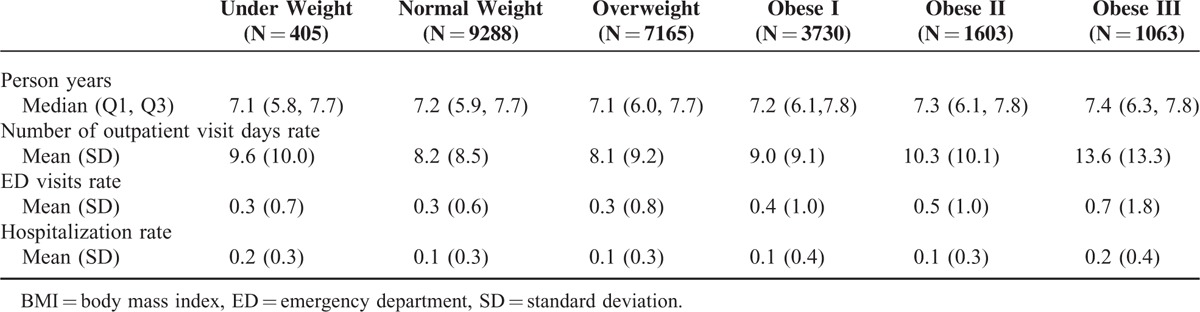
Rate for Outpatient Visit Days, Emergency Department Visits, and Hospitalizations by Baseline Body Mass Index Class

**TABLE 3:**
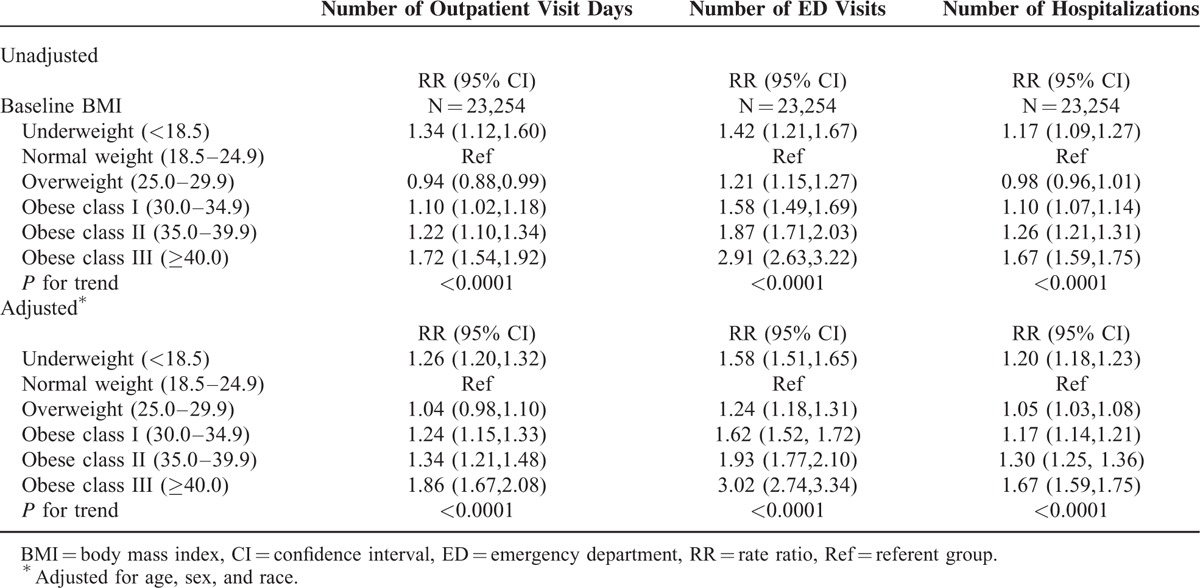
Rate Ratios for Outpatient Visit Days, Emergency Department Visits, and Hospitalizations by Baseline Body Mass Index

**FIGURE 1:**
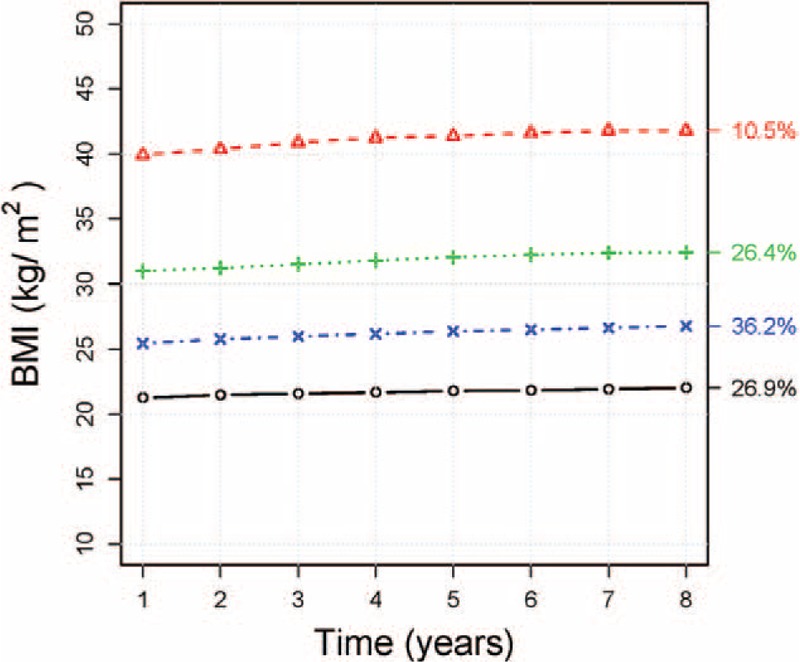
Body mass index (BMI) trajectory plots representing the mean BMI of all individual trajectories in a cluster combined for males and females. The percentages refer to the proportion of individuals in each trajectory. Trajectories: Normal, Overweight, Obese, Severely obese.

We next determined whether changes in BMI over time were associated with an increased risk of outpatient visit days, ED visits, and hospitalizations. We identified four latent-class BMI trajectories in our population (Figure 1). Overall, 26.9% of the population fell into a normal weight trajectory, 36.2% into an overweight trajectory, 26.4% into an obese trajectory, and 10.5% into a severely obese trajectory. Trajectories of BMI over time were very stable, with minor increases in BMI over 8 years as follows: normal weight trajectory (+0.77 kg/m^2^); overweight trajectory (+1.34 kg/m^2^); obese trajectory (+1.43 kg/m^2^); and severely obese trajectory (+1.87 kg/m^2^). Individuals in the overweight, obese, and severely obese trajectories were at a higher risk of frequent outpatient visits, ED visits, and hospitalizations after adjusting for age, sex, and race (Table 4). However, the increased risks of outpatient visit days and hospitalizations disappeared and the risk of ED visits was attenuated after further adjusting for baseline BMI levels; suggesting that baseline BMI accounted for much of the increased risk rather than changes in BMI over time (Table 4).

**TABLE 4:**
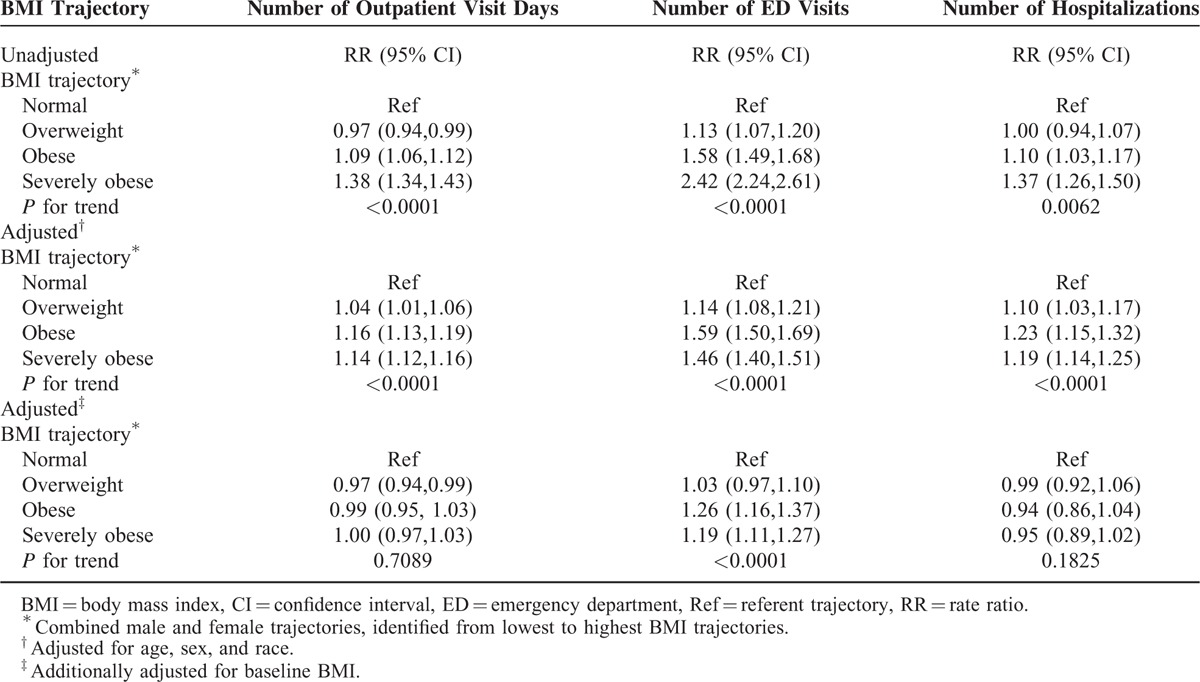
Rate Ratios for Outpatient Visit Days, Emergency Department Visits, and Hospitalizations by Body Mass Index Trajectory

## DISCUSSION

The findings of this study complement previous work examining the relationship between BMI trajectory and healthcare utilization in children,^25^ as well as, a study of self-reported recall of healthcare utilization over a 12-month period in German adults.^24^ We examined the association between BMI trajectories and healthcare utilization in young and middle-aged US adults. Baseline prevalence rates of obesity in our sample were comparable to national rates and trends by age and race.^2^ Adults in our study followed one of four BMI trajectories over an 8-year period with slight, progressive increases in BMI by each trajectory at the end of follow-up, ranging from 0.77 to 1.87 kg/m^2^ for the normal weight and severely obese trajectories, respectively. These findings are consistent with prior studies describing both the underlying heterogeneity and relative stability of BMI trajectories throughout adulthood, indicating that if individuals enter into adulthood as obese, they are likely to remain so with some weight gain as they age.^11,16,17,19,22,34^ In comparison to normal weight adults, we observed that increasing baseline BMI is positively associated with an increased frequency of outpatient visit days, ED visits, and hospitalizations, consistent with previous studies.^12–14^ We also observed that underweight BMI is associated with increased outpatient visit days, ED visits, and hospitalizations. This finding is consistent with the observation of higher medical costs in underweight adults in comparison to those with normal weight.^7^ Furthermore, the findings of our study complements earlier work in older adults demonstrating the association of underweight BMI with increased healthcare utilization.^35^ In addition, we found that BMI trajectory is positively associated with ED visits, even after adjustment for age, sex, race, and baseline BMI. The unique contribution of BMI trajectory, above and beyond baseline BMI, underscores the importance of examining the impact of life-course trends in BMI on healthcare utilization.

We observed that BMI trajectory is associated with increased ED visits, but not outpatient visit days or hospitalizations. Several factors may underlie this pattern of healthcare utilization. Obesity is associated with lower self-rated general health status,^36^ which may reflect underlying poor health due to obesity-related comorbidities and could predispose obese adults to seek care more often. However, stigmatization of obesity among healthcare providers may lead obese adults to delay or avoid obtaining timely and necessary healthcare.^37^ Obese adults also tend to receive fewer age and gender-specific preventative health services compared to nonobese individuals,^37,38^ which can lead to needing to manage complex, chronic health issues over time. Collectively, these factors disrupt high value continuity of care with primary providers that can lead to inefficient healthcare utilization such as more frequent ED visits.^39^ Furthermore, 4 of the top 5 indications for hospitalization among US adults (aged 18–44 years) are pregnancy-related conditions,^40^ which may explain why we did not find an association between BMI trajectory and hospitalization in this age group. It may also be that 8 years of follow-up at this stage of adulthood is both too short and too early to detect divergent patterns of healthcare utilization by BMI trajectory. Future study should seek to explore whether the association between BMI trajectory and healthcare utilization persists in an older cohort where chronic, obesity-related conditions are more prevalent.

Strengths of our study include a large sample size and accessibility to data for a defined population through the REP. As a result, we benefitted from effective capture of health service use as well as measured BMI in the EHR rather than reliance on self-reported data. Our study is representative of young and middle-aged adults residing in the upper Midwest^30^ and the prevalence of obesity observed was comparable to national rates based on age and race.^2^ However, additional studies in populations with strong ethnic or socioeconomic determinants may be needed to confirm that these results are fully generalizable across the country.^30^

Our study was limited to data available in the EHR, some of which may be missing or have been incorrectly entered. However, errors such as these are likely to be small, random, and few so as not to exert systematic effects of bias. BMI data were available for only 58% of the target population and trajectory analysis was based only on those with at least 3 BMI measures (72% of those with BMI data). Because BMI data were available only if a subject utilized health services for it to be recorded, it is possible that our analyses may be biased compared to adults for whom we did not have BMI data. Olmsted County residents receive health services from relatively few healthcare providers and we anticipated BMI data for at least 30% of our population would be unavailable due to limitations of records accessibility. However, we did not believe that BMI or patterns of utilization would differ considerably for Olmsted County residents receiving care solely at another local healthcare provider. Although the relative demographic comparability of our sample with the rest of Olmsted County suggests this to be true, this limitation must still be considered. Lastly, while our study delineates the underlying heterogeneity of BMI trajectories at a population level, heterogeneity also exists within each BMI category with potential implication for healthcare utilization and mortality as well.

In conclusion, we observed that baseline BMI and BMI trajectory influences healthcare utilization over an 8-year period in young- and middle-aged US adults. Over time, adults with higher BMI trajectories are likely to have increased ED utilization with its associated impact on cost and healthcare experience. Coupled with their comparatively lower receipt of overall healthcare services, obesity in early and middle adulthood represents a missed opportunity by the healthcare system to efficiently and effectively engage this population to reverse this chronic condition.
